# Clinical, histological and electron microscopic aspects of vocal fold granulomas

**DOI:** 10.1016/S1808-8694(15)30842-9

**Published:** 2015-10-18

**Authors:** Regina Helena Garcia Martins, Norimar Hernandes Dias, Daniela Carvalho dos Santos, Alexandre Todorovic Fabro, José Reinaldo Cerqueira Braz

**Affiliations:** 1 Livre docente certification professor in the Otorhinolaryngology Discipline, Faculdade de Medicina de Botucatu - Unesp. Faculty member of the Otorhinolaryngology Discipline.; 2 Otorhinolaryngologist, Otorhinolaryngology Discipline, Faculdade de Medicina de Botucatu, Unesp. Master’s degree in surgery. Graduate student, Otorhinolaryngology Discipline, Faculdade de Medicina de Botucatu-UNESP; 3 Assistant professor, doctor, Morphology Department, Instituto de Biociências - UNESP - Botucatu. Faculty member of the Morphology Department.; 4 Medical resident, Pathology Department, Faculdade de Medicina de Botucatu, UNESP; 5 Full professor of Anesthesiology, Faculdade de Medicina de Botucatu-Unesp. Faculty member of the Anesthesiology Department. Universidade Estadual Paulista Júlio de Mesquita Filho, Faculdade de Medicina de Botucatu-UNESP

**Keywords:** granuloma, intubation, larynx, morphology

## Abstract

Granulomas are bilateral and pediculated lesions of the vocal apophysis. Etiologies: intubation, reflux, trauma, vocal abuse, idiopathic origin. **Aim:** To analyze the clinical and morphological aspects of post intubation granulomas. **Methods:** retrospective study of patients submitted to microsurgery for post intubation laryngeal granulomas seen at our Medical School starting in 2002. We analyzed: age, gender, indication and time of intubation, symptoms, videolaryngoscopic diagnosis and biopsy findings. Light microscopy was performed on all specimens, and electron microscopy on three of them. **Results:** ten patients (7 females and 3 males), between the ages of 2 and 72 years, intubation time between 4h and 21 days. Hoarseness was a frequent symptom, starting in the first week following extubation. Histology shows mild epithelial hyperplasia, severe inflammation and vessel proliferation in the corion. Under SEM, the epithelium presented mild superficial desquamation. Under TEM, intracellular junctions showed widening with structural changes in the desmosomes. In the corion there were vessel proliferations, inflammation and fibroblasts with structural alterations. **Conclusions:** post intubation granulomas appear in any age and hoarseness is a frequent symptom. Morphological alterations occur in the corion as vessel proliferations, inflammation, and intracytoplasmatic alterations in fibroblasts suggesting cellular dysfunction and damage.

## INTRODUCTION

**Figure 1:**
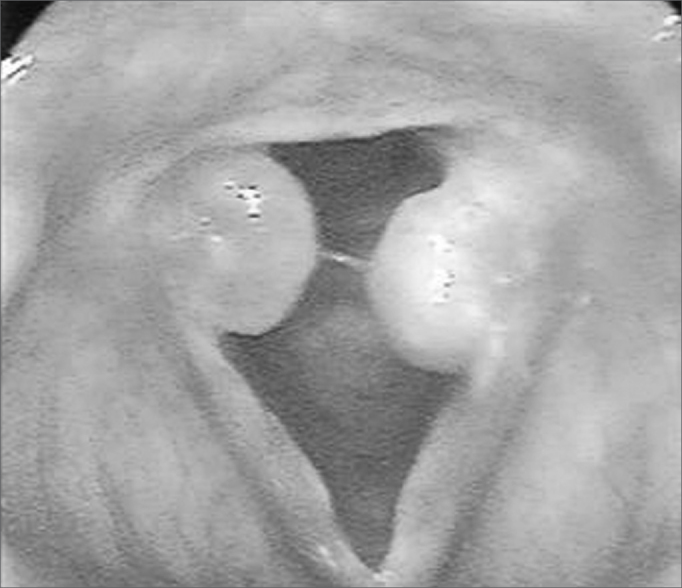
Granulomas in both vocal apophyses

Granulomas are unilateral or bilateral rounded lesions of various colors (rose, white or wine-color), with a pedicle most of the times, and a smooth or irregular surface. The implantation pedicles are inserted on the posterior area of the glottis, especially on the vocal apophysis (Figure 1).

The etiological factors causing the laryngeal granulomas include endotracheal intubation, gastroesophageal reflux, external laryngeal trauma, phonotrauma, and idiopathic origin, when the cause cannot be found. Among these etiological factors, endotracheal intubation, voice abuse and acid laryngitis are the most relevant, which underlines the need for investigating these possibilities in the clinical history.^1^, ^2^ Specific laryngeal granulomas, secondary to specific granulomatous diseases of the larynx, such as tuberculosis, blastomycosis, leprosy, leishmaniasis, syphilis, and other, should also be taken into account.^1^ This study will focus mainly the intubation granulomas.

In 1932, Clawsen first described laryngeal granulomas as a result of endotracheal intubation, raising the attention of other authors to the study of its predisposing factors.^3^ Among them, we have: prolonged and traumatic intubation, use of larger diameter tracheal tubes, high pressure inside the tracheal tube balloons especially in anesthesia using nitrous oxide, and inadequate sedation levels. In the latter, movement of the patient’s neck causes the tube to move within the airway, causing friction between the tube and the lining epithelium in the laryngeal and tracheal mucosa. Intubated patients that are inadequately sedated or not sedated at all perform involuntary swallowing and phonatory movements, pushing the vocal folds against the tube and compressing or traumatizing the lining epithelium of the vocal apophyses.^4^, ^5^.

Some authors have found a higher prevalence of laryngeal granulomas in males. This ratio is inverted when intubation granulomas are studied specifically; these are more common in the female larynx. This fact has been explained by the anatomical specificities of the female larynx, which is smaller compared to the male larynx, thus allowing more contact between the cannula and the airway mucosa.^1^, ^6^ Furthermore, the fragile perichondrium that covers the vocal apophysis of the arytenoid cartilages and the poor circulation of the local mucosa are additional factors that make the area more vulnerable to intubation trauma. The prevalence of intubation granulomas in females was confirmed by Pontes et al.^7^ in an analysis of 66 patients with laryngeal granulomas, 15 of which developed after intubation; six of these were male and nine were female.

In many cases voice is not affected, since the granulomas are located on the posterior glottic commissure, and glottic coaptation is not affected. However, there have been descriptions of large granulomas that not only affect voice quality considerably, but also cause dyspnea.^8^ Additional symptoms include a foreign body sensation, coughing and phlegm.

Intubation granulomas occur more often after prolonged intubation, although they have been diagnosed in patients intubated for short periods. Kaneda et al.^9^ reported a vocal fold granuloma in a female patient that was intubated with a small diameter tracheal tube (6.5 mm) for only 4.5 hours. Shimokojin et al.^10^ described shorter intubation times resulting in laryngeal granulomas in three patients (2 hours and 20 minutes to 5 hours and 40 minutes.

Few authors have described the morphology of laryngeal granulomas. The rare descriptions are restricted to non-ultrastructural histological studies.^1^, ^11^ Luzar et al.^11^ undertook a retrospective, clinical and histomorphological study of the epithelial features of the mucosal lining on 149 laryngeal granulomas. These authors applied the Ljubljana^12^ classification in the histological analysis of epithelial alterations, which describes epithelium as normal, simple and abnormal hyperplasia, atypical hyperplasia, and in situ carcinoma. An analysis of epithelial atrophy completed the study. The authors underlined the benign features of laryngeal granulomas based on the finding of simple epithelial hyperplasia in 65.8% of cases, epithelial atrophy in 16.1% of cases, a normal epithelium in 13.4% of cases, and abnormal hyperplasia in only 4.7% of cases; they found no in situ carcinomas. The authors also found many pools of blood permeated with inflammatory cells in the lamina propria.

More detailed morphological descriptions of intubation granulomas were not found in the literature, which motivated the present paper. Its purpose is to present the clinical and morphological features of intubation granulomas, done by histological studies, and transmission and scanning electron microscopy, to show ultra-structural details that might increase our understanding of the pathophysiology of granulomas and guide the treatment.

## SERIES AND METHOD

A retrospective study was undertaken of patients with laryngeal granulomas due to endotracheal intubation, seen at the outpatient otorhinolaryngology units of the Botucatu Medical School (Faculdade de Medicina de Botucatu, Unesp) from 2002 onwards, and that had undergone laryngeal microsurgery for removing the lesions. The Research Ethics Committee of the institution above in which research was undertaken approved the study (protocol number 472/2007).

The following information was taken from medical files: age, sex, indication for intubation, duration of intubation, voice or respiratory symptoms manifested after extubation, videolaryngoscopy reports, and biopsy number.

A qualified professional from the Pathology Department of the aforesaid institution reviewed the histological slides, and recorded the most relevant epithelial changes (hyperplasia, acanthosis, hyperkeratosis) and those in the lamina propria (increased number of vessels, edema, inflammatory cell infiltrate). Histological parameters were quantified based on a semi-quantitative score: 0 (unaltered), 1 (mildly altered), 2 (moderately altered) and 3 (intensely altered). A light microscope (Axiostar plus model, Zeiss, Carl Zeiss do Brasil Ltda) was used for examination and photography at different magnifications; a digital camera was used for recording the images.

An additional three fragments of laryngeal granulomas had been fixated in 2.5% glutaraldehyde at the time of surgery and sent to the Morphology Department of the same institution for processing and electron microscopy; the procedures are described next. For scanning electron microscopy, the surgical specimens were fixated in 2.5% glutaraldehyde during 12 hours, the washed in 0.1 M buffered phosphate at pH 7.3, fixated in a 1% osmium tetroxide solution during 1 hour, washed in buffered phosphate, dehydrated in series in alcohol solutions (75% to 100%), then dried in a critical point dryer device (Balzers CPD-020) with liquid carbon dioxide. The specimens were then mounted on a metal base using silver glue and gold-covered (15 nm of gold) in a Balzers MED-010 mini deposition device. A scanning electron microscope (SEM 515, Philips, Netherlands) at 15 KV was used for examining and photographing the specimens. For transmission electron microscopy, the specimens were fixated in 2.5% glutaraldehyde, washed in 0.1 M buffered phosphate at pH 7.3, sectioned into 3 mm x 1 mm specimens over pink dental wax humidified with a 2.5% glutaraldehyde fixating solution, then fixated in a 1% osmium tetroxide solution and 0.1 M buffered phosphate at pH 7.3, dehydrated in series in acetone solutions (50%, 70%, 90% and 100%), and placed in a mixture of acetone and araldite resin (Polysciences, Inc.). The fragments were then removed from this mixture and included in a pure araldite resin block in an oven at 37°C; semifine 0.5 μm sections were made, placed on slides and stained with a 1:1 mixture of 1% methylene blue and 1% Azur II. These semifine sections were examined in a light microscope and then again sectioned into ultrafine specimens (500), and examined and photographed in a transmission electron microscope (EM 301 model, Philips AG, Netherlands), using an Eastman 5302 (Kodak Co, US) film and Kodabromide (Eastman Kodak Co., US) photographic paper.

We had already defined the normalcy standards for the laryngeal mucosa for both histology and electron microscopy in previous studies. These were based on a morphological analysis of the vocal fold mucosa removed from three autopsy laryngeal specimens with no history of intubation or laryngeal trauma.

The chi-square test was used for comparing the proportions. Friedman’s non-parametric test was used for comparing the scores. The significance level was 5 %.

## RESULTS

Within the study period, intubation granulomas were removed from 10 patients; Table 1 shows their clinical features. The age of patients ranged from 2 years and 3 months to 72 years; there were mostly female patients (7 cases). The intubation time ranged from 4 hours to 21 days. The main symptom was hoarseness, which in most cases started during the first week after extubation; in some cases, hoarseness was present immediately after removal of the cannula. The most frequent endoscopic finding was bilateral granulomas in the vocal apophysis. Recurrence of the lesions was seen in one patient only.

**Table 1:** Clinical findings inpatients with intubation granulomas

Case	Age	Sex	Cause of intubation	Duration of intubation	Symptoms	Time from onset of symptoms and extubation	Laryngoscopy	Relapses
1	23	F	Laparoscopy	6 hs	Hoarseness and coughing	12 days	Bilateral granulomas	3
2	53	F	Facial plastic surgery	4 hs	Hoarseness	7 days	Bilateral granulomas	0
3	2a 3m	F	Pneumonia	21 days	Hoarseness and dyspnea	2 days	Granuloma anterior + subglottic stenosis	0
4	23	M	Cranial trauma	12 days	Hoarseness	3 days	Bilateral granulomas	0
5	16	F	Over dose	3 days	Hoarseness	30 days	Bilateral granulomas	0
6	20	F	Post-cesarean complications	1 dia	Hoarseness	1 h	Granuloma unilateral post	0
7	4 a	F	Seizures	1 dia	Hoarseness	1 h	Granuloma unilateral	0
8	72	M	Angioplasty	3 days	Hoarseness	60 min	Bilateral granulomas	0
9	37	M	Cranial trauma	2 days	Hoarseness	5 days	Bilateral granulomas	0
10	29	F	Cranial trauma	7 days	Hoarseness	15 days	Bilateral granulomas	0

**Figure 2:**
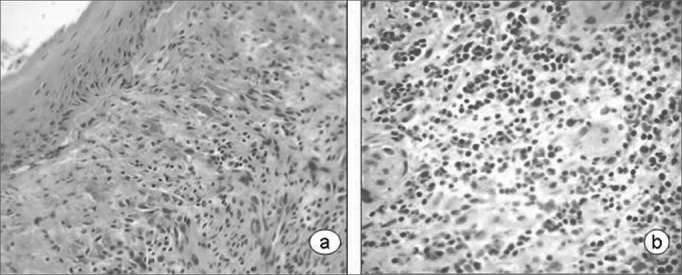
Laryngeal granuloma. Marked inflammatory cell infiltrate in the lamina propria in a and b; proliferation of small vessels (light microscopy, HE- a-40X; b-80X).

Table 2 summarizes the histological results; the epithelium was mildly altered, mostly mild hyperplasia recorded in seven slides. The lamina propria was more deeply altered; there was an inflammatory infiltrate of polymorphonuclear cells, lymphocyte and occasional histiocytes (Figure 2). Intense proliferation of vessels was a constant finding in the slides (Figure 2). Table 2 highlights (*) the histological changes that were statistically significant (p<0.05) in the comparison of scores.

**Table 2:** Histological analysis of intubation granulomas

Case	EPITHELIUM				LAMINA PROPRIA			
	Hyperplasia (*)	Acanthosis	Hyperkeratosis	Edema (*)	PMN (*)	Lymphocytes (*)	Histiocytes	Proliferation of vessels (*)
1	0	0	0	1	1	2	0	3
2	0	0	0	2	1	1	1	3
3	1	1	0	2	1	2	0	3
4	0	0	0	2	2	1	0	3
5	1	0	0	3	1	1	0	2
6	1	1	0	2	1	1	0	1
7	1	0	0	2	1	3	0	1
8	1	0	1	3	0	1	0	1
9	1	0	0	2	2	1	0	2
10	1	0	0	2	2	1	0	2

Score system: 0 - unaltered; 1 - mildly altered; 2 - moderately altered; 3 - severely altered.

(*) p<0,05

**Figure 3:**
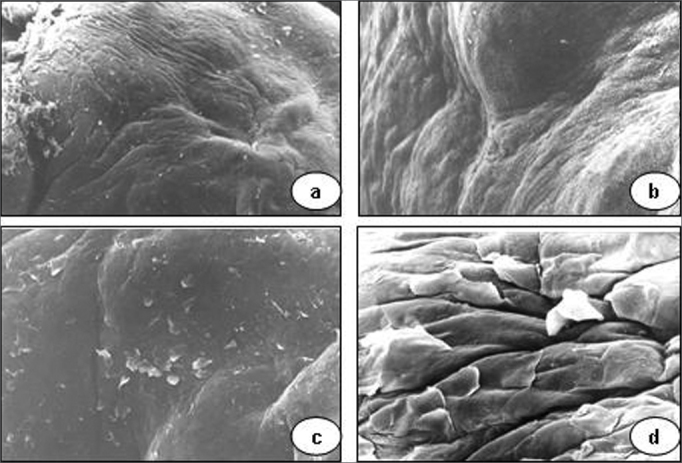
Vocal granuloma in a, b and c. Note the smooth surface of the mucosa and few desquamating cells (scanning electron microscope: a - 55 X; b - 330 X; c - 165 X). In d (890 X), the mucosal surface of a normal vocal fold showing the hexagonal pattern and some cells detaching from the surface.

Scanning electron microscopy of granulomas revealed a squamous epithelium with few desquamating cells from its surface, the opposite of normal plicated and exfoliative epithelium of vocal folds (Figure 3).

**Figure 4:**
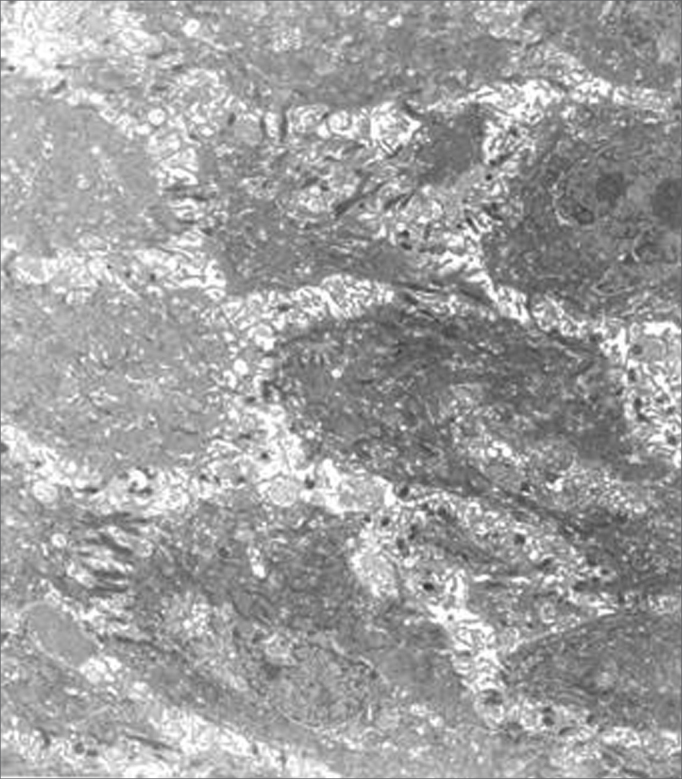
Epithelial surface of a laryngeal granuloma; widening of cell junctions, changing the structure of desmosomes (transmission electron microscope, 1 550 X).

**Figure 5:**
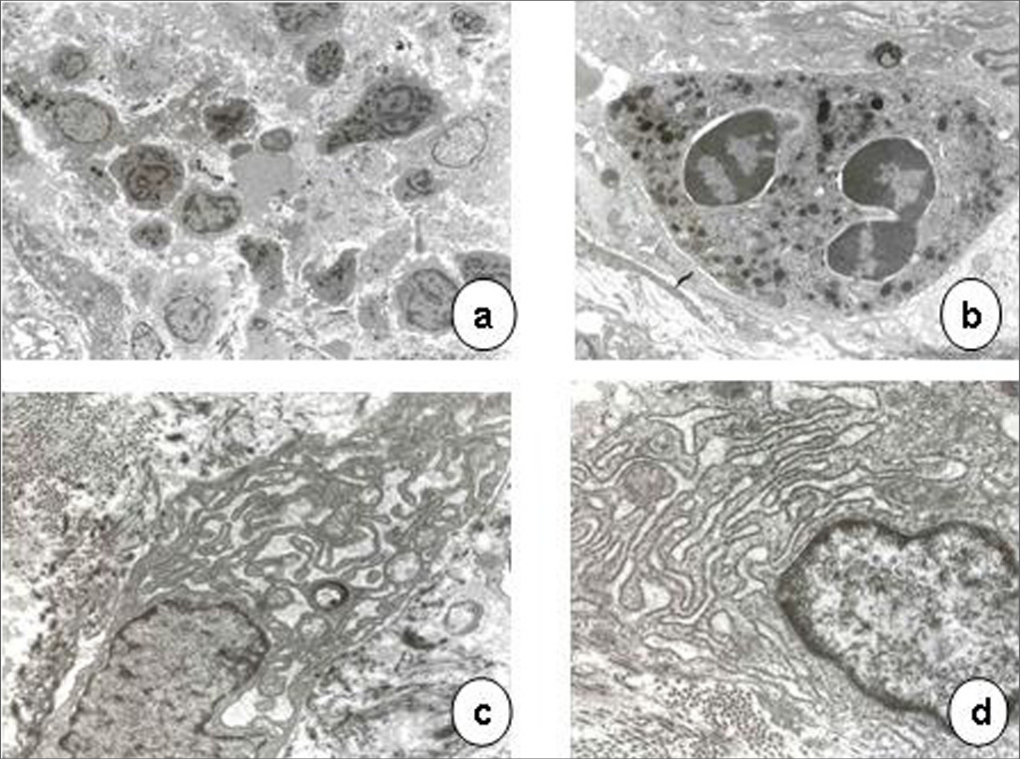
Laryngeal granuloma. In a, an intense inflammatory cell infiltrate in the lamina propria amid amorphous material (3 250 X); in b, highlighting a polymorphonuclear neutrophil with its cytoplasm full of phagocytosed granules (13 250 X). In c and d, fibroblasts with altered morphology, containing rough endoplasmic reticulum with extremely dilated cisterns and irregularly contoured nuclei (13 250 X).

**Figure 6:**
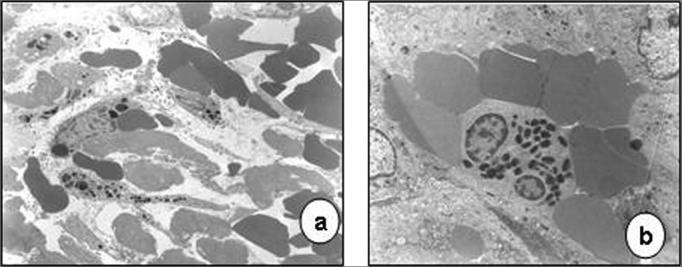
Laryngeal granuloma. In a, lamina propria with pools of blood formed by vascular proliferation (7 700 X). In b, highlighting an eosinophil with birefringent cytoplasmatic granules (7 700 X).

Transmission electron microscopy showed that epithelial cell junctions were different among themselves, with altered desmosome structures (Figure 4). Many inflammatory cells, especially neutrophils, lymphocytes, some eosinophils and mast cells were found in the lamina propria (Figures 5 and 6). The cytoplasm of some neutrophils and mast cells were filled with phagocytosed granules. Pools of blood were also seen in the lamina propria (Figure 6). Fibroblasts were more numerous in the lamina propria, and exhibited markedly altered cytoplasm, with dilatation of endoplasmic reticulum cisterns and irregularly contoured nuclei (Figure 5). There were no collagen fiber networks, notwithstanding the increased number of fibroblasts.

## DISCUSSION

A variety of laryngeal and tracheal lesions secondary to endotracheal intubation have been described, including ulcers of the mucosa, synechiae, stenosis, and granulomas.^4^, ^5^ The common pathophysiological condition in these lesions is ischemia of the mucosa, especially due to wider diameter endotracheal tubes and high cuff pressures. Along these lines, Nordin^13^ undertook an interesting experimental study of intubated rats in which he varied the balloon pressure from 20 to 100 mmHg, finding that lesions were directly related with increased pressure in the balloon, particularly when above the capillary perfusion pressure (25 mmHg). This led, in sequence, to ischemia of the mucosa, loss of blood supply to the perichondrium, local ulcers, secondary bacterial colonization, and chondritis. The voice process area is not directly in contact with the balloon, but rather with the cannula; given its thin mucosal and perichondrial layer, it is frequently injured during intubation, particularly in traumatic intubation and restless patients.

Granulomas are mostly described in adult patients with prolonged intubation. In our study, to the contrary, we found that these injuries can also occur in the infant larynx; we found them in a child aged 2 years and 3 months. The intubation time does not seem to be the only determining factor for injury; Table 1 shows data of some patients that developed granulomas after just a few hours of intubation time, a fact that other authors have highlighted.^9^, ^10^

Granulomas are implanted on the posterior portion of the glottis; as such one would not expect them to cause voice symptoms immediately after extubation, since they to not develop immediately. However, complaints of hoarseness are frequent, and often arise soon after extubation or in the next few days. The reason whereby some patients develop granulomas and voice symptoms early remains uncertain. Other factors appear to operate, such as gastroesophageal reflux and abuse of voice, which are also the main causes of laryngeal granulomas. Pontes et al.^7^ studied 66 patients retrospectively and found that the main causes of laryngeal granulomas were: gastroesophageal reflux (30.3%), abuse of voice (33.3%), endotracheal intubation (22.7%), and of idiopathic origin (9%). Wani & Woodson^3^ also highlighted gastroesophageal reflux in the origin of laryngeal granulomas; it was diagnosed in 19 of 21 patients. Endotracheal intubation was a less frequent cause in their series (four patients). Lack of additional information in the medical files did not allow us to analyze the influence of this predisposing factor. The habit of coughing and clearing the throat in the postoperative period also traumatizes the laryngeal mucosa. Thus, these actions should be contained after extubation to avoid lesions from developing. Lemos et al.^2^ demonstrated the importance of controlling gastroesophageal reflux, inflammation, and voice abuse in the treatment of laryngeal granulomas, regardless of their origin. These authors attained significant remission of granulomas after medical therapy during 2 to 4 months; treatment included using proton pump inhibitors, inhaled corticosteroids and speech therapy. Recurrences noted by many authors and observed in one of our cases may be due to poor control of those factors. Relapses often occur early; they have been controlled by surgical treatment of granulomas associated with type A botulin toxin injections into the thyroarytenoid muscle to decrease the impact between vocal folds during phonatioin.^7^, ^14^

The morphological findings we observed showed that the epithelium in intubation granulomas is squamous, that there is mild epithelial hyperplasia and edema, which was detected in transmission electron microscopy as the distancing of cell junctions. Widening of epithelial cell junctions and epithelial hyperplasia are often highlighted in ultrastructural studies of other benign vocal fold lesions, such as nodules, polyps, and Reinke’s edema. In these cases, the epithelium is constantly exposed to trauma from abuse of voice, smoking, inflammation, and others.^15^, ^16^

Epithelial erosion or neoplastic features were not found in any of the histological and ultrastructural samples. Galé et al.^12^ had already underlined the benign nature of granulomas in a retrospective study of 149 biopsies of laryngeal granulomas; these authors showed that simple epithelial hyperplasia, atrophy and normal epithelial predominated.

The lamina propria of normal vocal folds consists basically of extracellular matrix containing collagen fibers, elastic fibers, glycoproteins, glycosaminoglycans, some fibroblasts, occasional mast cells, and sparse capillaries.^17^, ^18^ An intense inflammatory cell infiltrate with proliferation of blood vessels and a predominance of polymorphonuclear neutrophils, lymphocytes, occasional histiocytes and eosinophils was found in the lamina propria in ultrastructural and histological studies of granulomas. These changes characterize granulomas as an inflammatory angiomatous lesion, also called pyogenic granuloma. The cytoplasm of many of these cells was filled with phagocytosed granules, suggesting marked inflammation repair activity.

Fibroblasts are usually numerous in damaged tissues; they help repair tissues by producing collagen fibers. In granulomas on the other hand, collagen fibers are not seen in the lamina propria, even with an increased number of fibroblasts. Ultrastructural analysis of these cells revealed significant intra-cytoplasmatic changes, such as dilated rough endoplasmic reticulum and loss of ribosomes. These changes, together with irregularly contoured nuclei, are interpreted as cell dysfunction and damage.

The predominant inflammatory component in granulomas explains the benefits of adjuvant therapy with corticosteroids.

## CONCLUSION

Post-endotracheal intubation vocal granulomas may arise at any age and in patients subjected to short-term intubation. Voice symptoms appear early. Morphological analysis of granulomas shows marked proliferation of vessels in the lamina propria and intense inflammation. Additional ultrastructural studies reveal intra-cytoplasmatic changes in fibroblasts, suggesting cell dysfunction and damage.
